# Multiple forms of hotspots of tetrapod biodiversity and the challenges of open-access data scarcity

**DOI:** 10.1038/s41598-020-79074-8

**Published:** 2020-12-16

**Authors:** Florencia Grattarola, Juan A. Martínez-Lanfranco, Germán Botto, Daniel E. Naya, Raúl Maneyro, Patricia Mai, Daniel Hernández, Gabriel Laufer, Lucía Ziegler, Enrique M. González, Inés da Rosa, Noelia Gobel, Andrés González, Javier González, Ana L. Rodales, Daniel Pincheira-Donoso

**Affiliations:** 1 School of Life Sciences, University of Lincoln, Brayford Campus, Lincoln, LN6 7TS UK; 2 Department of Biological Sciences, Centennial Centre for Interdisciplinary Science Bldg., University of Alberta, Edmonton, AB T6G 2E9 Canada; 3 Departamento de Métodos Cuantitativos, Facultad de Medicina, Universidad de La República, Av. Gral Flores 2125, 11800 Montevideo, Uruguay; 4 Departamento de Ecología Y Evolución, Facultad de Ciencias, Universidad de La República, Iguá 4224, 11400 Montevideo, Uruguay; 5 Laboratorio de Sistemática e Historia Natural de Vertebrados, Facultad de Ciencias, Universidad de La República, Iguá 4224, 11400 Montevideo, Uruguay; 6 Departamento de Ecología Y Gestión Ambiental, Centro Universitario Regional del Este (CURE), Universidad de La República, Tacuarembó s/n, 20000 Maldonado, Uruguay; 7 Polo Educativo Tecnológico Arrayanes (CETP-UTU), Camino Los Arrayanes s/n, 20200 Piriápolis, Uruguay; 8 Área Biodiversidad Y Conservación, Museo Nacional de Historia Natural, 25 de Mayo 582, 11000 Montevideo, Uruguay; 9 Museo Nacional de Historia Natural, 25 de Mayo 582, 11000 Montevideo, Uruguay; 10 Parque Tecnológico de LATU, Av Italia 6201, Universidad Tecnológica del Uruguay, 11500 Montevideo, Uruguay; 11 MacroBiodiversity Lab, School of Biological Sciences, Queen’s University Belfast, 19 Chlorine Gardens, Belfast, BT9 5DL UK

**Keywords:** Zoology, Macroecology, Biodiversity, Conservation biology

## Abstract

The uneven spatial distribution of biodiversity is a defining feature of nature. In fact, the implementation of conservation actions both locally and globally has progressively been guided by the identification of biodiversity ‘hotspots’ (areas with exceptional biodiversity). However, different regions of the world differ drastically in the availability of fine-scale data on the diversity and distribution of species, thus limiting the potential to assess their local environmental priorities. Within South America—a megadiverse continent—Uruguay represents a peculiar area where multiple tropical and non-tropical eco-regions converge, creating highly heterogeneous ecosystems, but where the systematic quantification of biodiversity remains largely anecdotal. To investigate the constraints posed by the limited access to biodiversity data, we employ the most comprehensive database for tetrapod vertebrates in Uruguay (spanning 664 species) assembled to date, to identify hotspots of species-richness, endemism and threatened species for the first time. Our results reveal negligible spatial congruence among biodiversity hotspots, and that tetrapod sampling has historically concentrated in only a few areas. Collectively, our study provides a detailed account of the areas where urgent biodiversity monitoring efforts are needed to develop more accurate knowledge on biodiversity patterns, offering government and environmental bodies a critical scientific resource for future planning.

## Introduction

The prevailing spatial unevenness in the distribution of biodiversity globally^1,2^ imposes pressing challenges to the understanding and conservation of ecosystems given that the distribution of threats is also spatially asymmetric^3–6^. Therefore, in an era of alarming worldwide biodiversity declines^7–10^, where the majority of species still remain to be discovered^11^, it is crucial to accurately identify the geographic regions of primary conservation concern^12,13^. Hotspots of biodiversity—areas characterised by exceptional relative species-richness, or by unusually high numbers of endemic and endangered species^14,15^—have largely been considered primary targets for such conservation actions^16–19^. Ecological criteria such as vulnerability^20,21^, irreplaceability^22^ and representativeness^23^ have been used to identify biodiversity areas of priority concern^17,24,25^. However, a major challenge intrinsic to studies of biodiversity distributions at large spatial or taxonomic scales is the difficulty that assembling comprehensive datasets that cover high proportions of the diversity of entire regions or clades involves^26,27^ (often referred to as ‘the Wallacean shortfall’^28^). This challenge is aggravated in some countries that tend to concentrate less comprehensive biodiversity datasets^29^ (i.e., data-poor regions), whilst often hosting the biodiversity-richest regions and undergoing the toughest pressures on biodiversity^28,30,31^.

Over the last 2 decades, increased efforts have been made to expand the spatial and/or taxonomic coverage of biodiversity datasets, with the aim to advance our ability to quantify biodiversity patterns and to make evidence-based decisions about conservation actions^18,32–35^. As comprehensive biodiversity datasets become available, evidence has revealed, among other core findings, that different measures of biodiversity hotspots are not consistently congruent in space^36,37^. Additionally, conservation actions are not effective across or even within the same clades, given the biological idiosyncrasies of each group^32^—for example, given that ectothermic tetrapods (amphibians, reptiles) do not experience the same environmental pressures that endothermic organisms (mammals, birds) do, their biological responses to the same environments can differ importantly^38^. The spatial and time scales considered are also expected to impact on the patterns being observed, and thus, finer-scale data tend to offer better resources for more effective inferences about the spatial organisation of biodiversity^39,40^. Therefore, efforts devoted to create truly comprehensive primary biodiversity datasets are expected to hold a vital key to understand and manage biodiversity^41,42^.

Within South America—one of the world’s most biodiverse continents^14,16^–, Uruguay, which spans one of the world’s most extensive natural grasslands^43,44^, stands-out given the convergence of tropical and non-tropical eco-regions that combined bring together the geographic boundaries of an important ‘mix’ of species representative of those major biological domains^45–50^. Yet, Uruguay’s network of protected areas spans < 1.5% of the country’s territory^51^—the lowest for any country in South America^52,53^. A dominant reason for such discrepancies between the country’s diversity and conservation actions can be attributed to the limited knowledge on the distribution of its biodiversity. While previous attempts to identify the geographic location of Uruguay’s biodiversity hotspots have been made^54–57^, the quantification of multiple forms of biodiversity hotspots remains fundamentally neglected given the severe gaps in geographic data and the lack of comprehensive open-access databases on species distributions. Thus, despite its unique ecosystem and biodiversity attributes, the availability of scientific tools for evidence-based management and longer-term planning of Uruguay’s environments lags behind most countries in the continent. To address this historical issue, Biodiversidata—Uruguay’s first Consortium of Biodiversity Data (https://biodiversidata.org/) has recently been created^58,59^. This initiative gathers a collaborative scientific community of the country’s biodiversity experts with the aim of assembling a constantly growing database for Uruguay’s biodiversity. The first version of Biodiversidata presented a comprehensive dataset for all tetrapod species^58^.

In this study, we investigate for the first time the distribution, diversity and congruence of Uruguay’s hotspots of tetrapods, using > 69,000 geographic records for 664 species of amphibians, reptiles, birds and mammals (Table 1, Supplementary Fig. S1) compiled as part of the emerging Biodiversidata initiative^58^. We aim to (1) elucidate the spatial patterns of tetrapod species-richness, endemism and threatened species (both measured as absolute and relative numbers of species facing extinction risk), and quantitatively assess their spatial congruence, (2) measure the sampling bias and evaluate estimated diversities, (3) assess whether the current protected area network in Uruguay is capturing the different hotspots, and (4) identify areas where future sampling to foster robust environmental management and planning should be prioritised.

**Table 1:** Occurrence records and unsampled area (grids without records) per tetrapod group. Number of records (non-duplicated records/location/year), number of species and unsampled area (expressed in km^2^ and percentage of the total area of Uruguay), for all tetrapods and separated classes, according to each grid-cell size (12.5 × 12.5 km, 25 × 25 km and 50 × 50 km).

	Number of records	Number of species	Unsampled area					
			12.5 × 12.5 km		25 × 25 km		50 × 50 km	
			km^2^	%	km^2^	%	km^2^	%
Tetrapods	69,364	664	54,527.2	30.9	10,792.7	6.1	10.6	0.01
Amphibians	2546	50	129,581.1	73.5	68,674.9	39	11,822.4	6.7
Reptiles	2343	68	97,163.5	55.1	40,172.5	22.8	7995.6	4.5
Birds	60,524	430	110,803.4	62.9	52,008.1	29.5	10,778.2	6.1
Mammals	3951	116	105,173.6	59.7	36,144.6	20.51	10.6	0.01

## Results

### The spatial distribution of biodiversity

**Figure 1:**
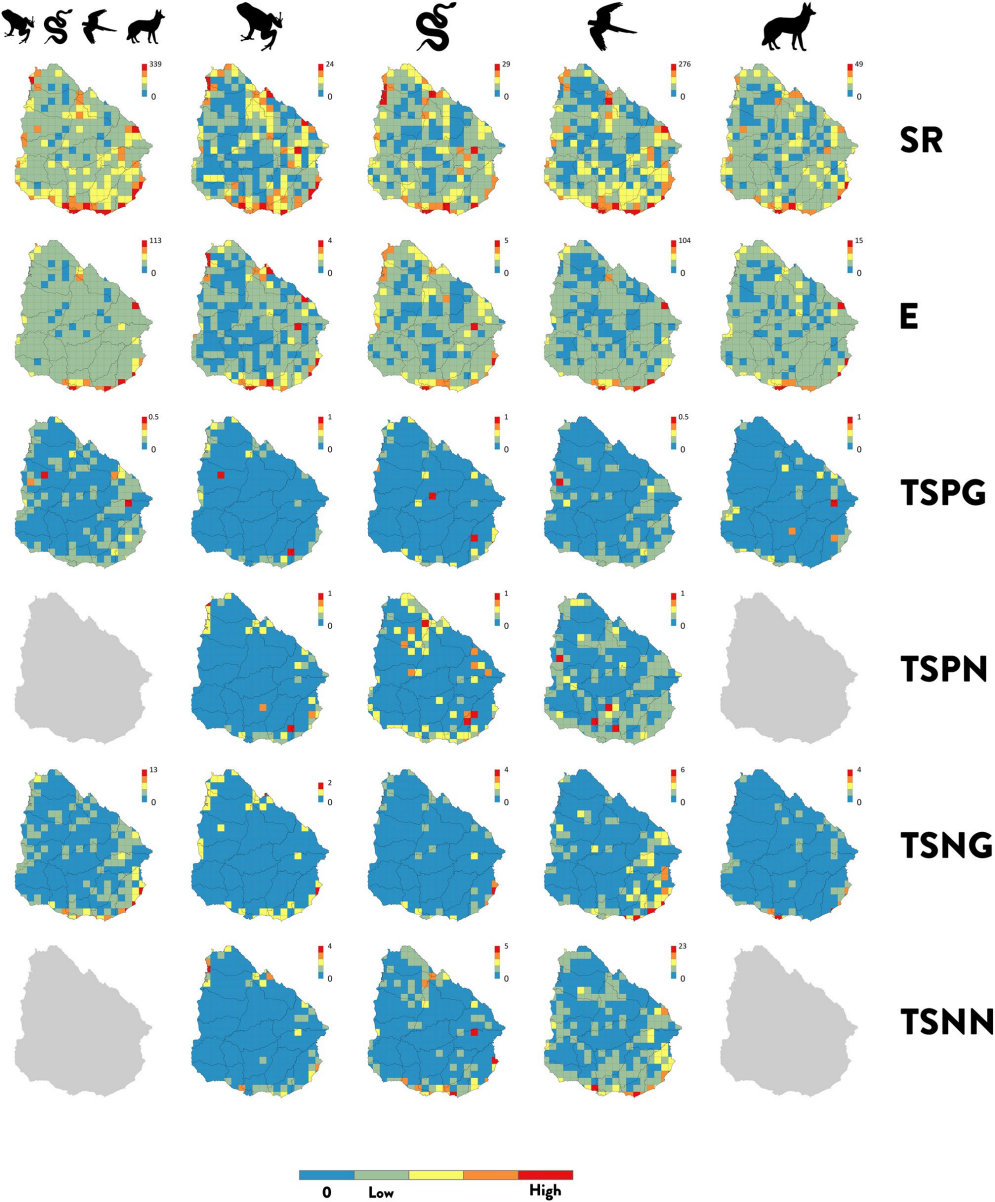
Spatial distribution patterns of hotspots of tetrapod species in Uruguay. Hotspots metrics for all tetrapods, amphibians, reptiles, birds and mammals (from left to right), of species-richness (SR) (top row), endemism (E) (second from the top row), threatened species proportion using the global IUCN assessment (TSPG) (third row) or national IUCN assessment (TSPN) (forth row), and threatened species number using the global IUCN assessment (TSNG) (fifth row) or national IUCN assessment (TSNN) (bottom row). Blue cells indicate either non-sampled areas or zero values of threatened species for both relative and absolute estimates. Because of lack of mammals’ threat assessment, values for mammals and tetrapods at national level could not be computed (grey maps are shown). Scale bar values differ between panels depending on the hotspot’s metric, top values are shown for each. All maps in 25 × 25 km grid-cell resolution. Projection WGS1984. Maps generated using ArcGIS 10.6 (https://desktop.arcgis.com).

The distributional patterns of all three types of hotspot (species-richness, endemism and threatened species number/proportion) varied across taxa (Fig. 1 and Supplementary Fig. S2,S3). Species-richness for all groups of tetrapods was aggregated in the south and south-east coast of the country (Fig. 1, top row), with the highest numbers of species detected in the surroundings of Montevideo (capital city) and other coastal cities. High numbers of species were also detected in the mid-east border with Brazil and in the north-west side of the Uruguay River. Each group also exhibited high concentrations of species in exclusive grid-cells—not shared between groups (Fig. 1, top row). Endemism patterns were more homogeneously distributed for all tetrapods, with moderate values all across the territory and few high endemism areas towards the south and south-east coast, north-west and the mid-east borders of the country (Fig. 1, second row from the top). For each separate tetrapod class, we detected a similar pattern. Amphibians presented exclusive areas of endemism in the northwest, northeast and mid-east of the country, birds in the south coast, and mammals in the south-east Atlantic coast. We did not observe any reptile-exclusive areas of endemism. The hotspots of threatened species for all tetrapods combined and separated exhibited a few grid-cells of high value, for both species proportions (Fig. 1, third and fourth rows) and numbers (Fig. 1, fifth and bottom rows), irrespective of the IUCN categories used to assess threatened species. However, when using national IUCN assessments, additional peak values were revealed. Each tetrapod class showed unique areas with a high proportion of threatened species which, contrary to species-richness and endemism, were principally located distant to the coast and towards the centre of the country. The scarce hotspots of threatened species identified in all cases are mainly due to the low number of species that are assessed as threatened (i.e., critically endangered, endangered and vulnerable), either globally with 4 species of amphibians (8% of the total species), 9 reptiles (13.2%), 15 birds (3.5%) and 8 mammals (6.8%), or at the national level, with 12 species of amphibians (24% of the total species), 8 reptiles (11.8%) and 40 birds (9.3%).

### The spatial congruence of biodiversity hotspots

**Figure 2:**
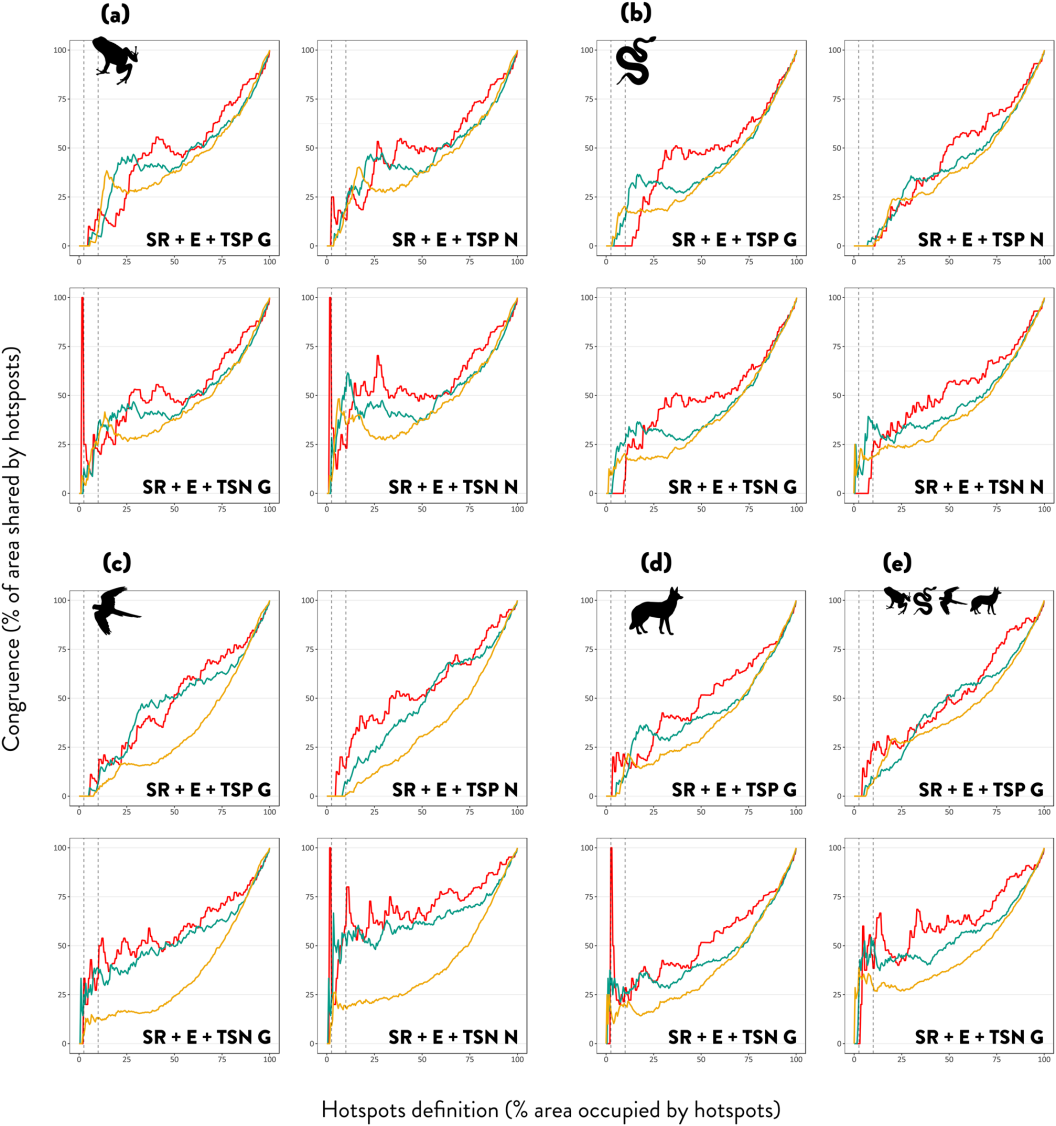
Extent of spatial congruence across types of hotspots. Species-richness (SR), endemism (E), threatened species proportion using the global IUCN assessment (TSP G) or national IUCN assessment (TSP N), and threatened species number using the global IUCN assessment (TSN G) or national IUCN assessment (TSN N). For amphibians (**a**), reptiles (**b**), birds (**c**), mammals (**d**) and tetrapods (**e**). Metrics are analysed with three different grid-cell sizes 50 × 50 km (red line), 25 × 25 km (green line) and 12.5 × 12.5 km (yellow line). Congruence is the number of cells that represent hotspots for all three diversity indices and is measured as the percentage of shared grid-cells over the percentage of land covered by hotspots according to a varying definition from 0 to 100% of the total grid-cells. Vertical dashed lines show 2.5% and 10% hotspot definition criterion.

We found a tendency for low spatial congruence across and within different measures of biodiversity hotspots when taking tetrapod classes combined or separately. The extent of spatial congruence across types of hotspots varied according to grid-cell size (12.5, 25 and 50 km), if threatened species proportion or number was considered, and the percentage of the hotspot area definition (% of area/number of cells occupied by hotspots; from 0 to 100%) (Fig. 2). However, we observed a tendency towards greater levels of congruence when relaxing the hotspot definition to 10% of the richest grid-cells, using the major grid-cell size and numbers of threatened species instead of proportions (Table 2a). Exceptionally for amphibians, birds and mammals, using the 50 × 50 km unit and considering the smallest definition of the area occupied by hotspots (2–2.5%), we observed complete congruence as the three types of hotspots were localised in the same unique grid-cell (Fig. 2a,c,d: red line). Amphibians and birds showed higher congruence when the national IUCN assessment was used (Fig. 2a,c). Yet, this was not the case for reptiles, which had a prevailing tendency for incongruence between diversity hotspots (Table 2a). The extent of spatial congruence of hotspots for tetrapods combined was generally higher than for each separate group (Fig. 2e).

**Table 2:** Hotspots congruence.

	Congruence (% of area shared by hotspots)						Analysis
	Grid-cell size						
	12.5 × 12.5 km		25 × 25 km		50 × 50 km		
	Hotspots definition		Hotspots definition		Hotspots definition		
	2.5%	10%	2.5%	10%	2.5%	10%	
**(a) Group**							
All tetrapods	0	6.7	0	8.8	0	26.7	Species Richness + Endemism + Threatened Species Proportion Global
Amphibia	0	8.1	0	5.1	0	18.8	
Reptilia	0	20.0	0	13.3	0	0	
Aves	0	3.7	0	6.8	0	6.2	
Mammalia	0	17.2	0	10.0	0	18.8	
All tetrapods	–	–	–	–	–	–	Species Richness + Endemism + Threatened Species Proportion National
Amphibia	0	18.6	0	18.2	25.0	13.3	
Reptilia	0	0	0	3.8	0	0	
Aves	0	0.9	0	6.7	0	14.3	
Mammalia	–	–	–	–	–	–	
All tetrapods	38.7	29.2	27.3	52.5	0	38.5	Species Richness + Endemism + Threatened Species Number Global
Amphibia	5.9	25.5	12.5	34.5	25.0	21.4	
Reptilia	11.5	20.0	0.0	26.2	0	6.7	
Aves	4.3	12.6	25.0	38.2	33.3	33.3	
Mammalia	16.7	19.6	30.0	25.6	100.0	28.6	
All tetrapods	–	–	–	–	–	–	Species Richness + Endemism + Threatened Species Number National
Amphibia	6.2	35.3	28.6	52.0	33.3	23.1	
Reptilia	14.8	18.9	15.4	33.3	0	21.4	
Aves	9.1	18.6	25.0	53.3	25.0	60.0	
Mammalia	–	–	–	–	–	–	
**(b) Type of hotspot**							
Species Richness	5.4	8.2	0	17.2	0	5.6	Amphibia + Reptilia + Aves + Mammalia
Endemism	13.6	15.1	0	13.6	0	5.3	
Threat. Sp. Proportion Global	0	0	0	1.8	0	0	
Threat. Sp. Number Global	0	2.6	0	14.7	0	4.8	

The extent of spatial congruence of (a) all types of hotspots considering all tetrapods and each tetrapod group and (b) each different type of hotspots considering all tetrapods together; varying both the size of the sampling unit (12.5x, 25 × and 50 × km) and the criterion to define a hotspots (%2.5 or 10% of area/number of cells occupied by hotspots).

Similarly, we observed very few overlapping grid-cells for each separate hotspot type when combining all tetrapods (Supplementary Fig. S4). Congruence increased by varying the hotspot definition criteria to the richest 10% of grid-cells, though it always remained less than 18% for all types of hotspots (Table 2b). The spatial overlap of hotspots of species-richness combining groups decreased with the increase in the grid-cell size, while the congruence of hotspots of endemism was less sensitive to it (Supplementary Fig. S4a, b). The congruence of hotspots of threatened species was very low regardless of grid’s size, for both species proportion and numbers (Supplementary Fig. S4c, d).

### Spatial correlation of sampling effort and biodiversity hotspots

The reconstruction of biodiversity hotspots critically depends on the historical distribution of sampling efforts. The spatial patterns of species-richness and endemism were significantly highly correlated with those of sampling effort for all tetrapod species and each single group (*r* ≥ 0.8, *p* < 0.001) (Supplementary Table S1). The spatial correlations between sampling effort and threatened species numbers were moderate to high for all groups (0.54 < *r* < 0.73, *p* < 0.001), while there were no significant correlations between threatened species proportions and sampling effort, or any metric of hotspot diversity.

Despite the lack of congruence across types of hotspots for each tetrapod group (Fig. 2), we saw moderate to high levels of spatial correlations between each pair of diversity measure (Supplementary Table S1), ranging from *r* = 0.706 to *r* = 0.802 (*p* < 0.001) for species-richness versus endemism, *r* = 0.521 to *r* = 0.793 (*p* < 0.001) for species-richness versus threatened species number, and *r* = 0.613 to *r* = 0.828 (*p* < 0.001) for endemism versus threatened species number, while there were no significant correlations between threatened species proportions and any other metric. For those groups with national red lists assessments (amphibians, reptiles and birds), we found higher correlations when using national IUCN threat categories rather than global, except in the case of reptiles’ spatial association between endemism and threat patterns (Supplementary Table S1).

When assessing how similarly distributed hotspot types between groups were, we found low to moderate correlation values for each type of diversity hotspot, all significant (*p* < 0.001) except when measuring threatened species proportions for any pair of tetrapod group (Supplementary Table S2). Species-richness patterns were moderately correlated, with amphibians versus reptiles showing the highest levels (*r* = 0.748), while the lowest were between amphibians and birds (*r* = 0.514, *p* < 0.001). Endemism revealed weaker associations between groups, ranging from *r* = 0.360 (*p* < 0.001) for amphibians versus birds, to a maximum of *r* = 0.697 (*p* < 0.001) between birds and mammals. The association between groups regarding the threatened species numbers showed similar low correlation values, below *r* = 0.595 (*p* < 0.001) for all pairs of classes analysed using global threat categories, and under *r* = 0.467 (*p* < 0.001) when using the national ones.

### Comparing true diversities among groups

**Figure 3:**
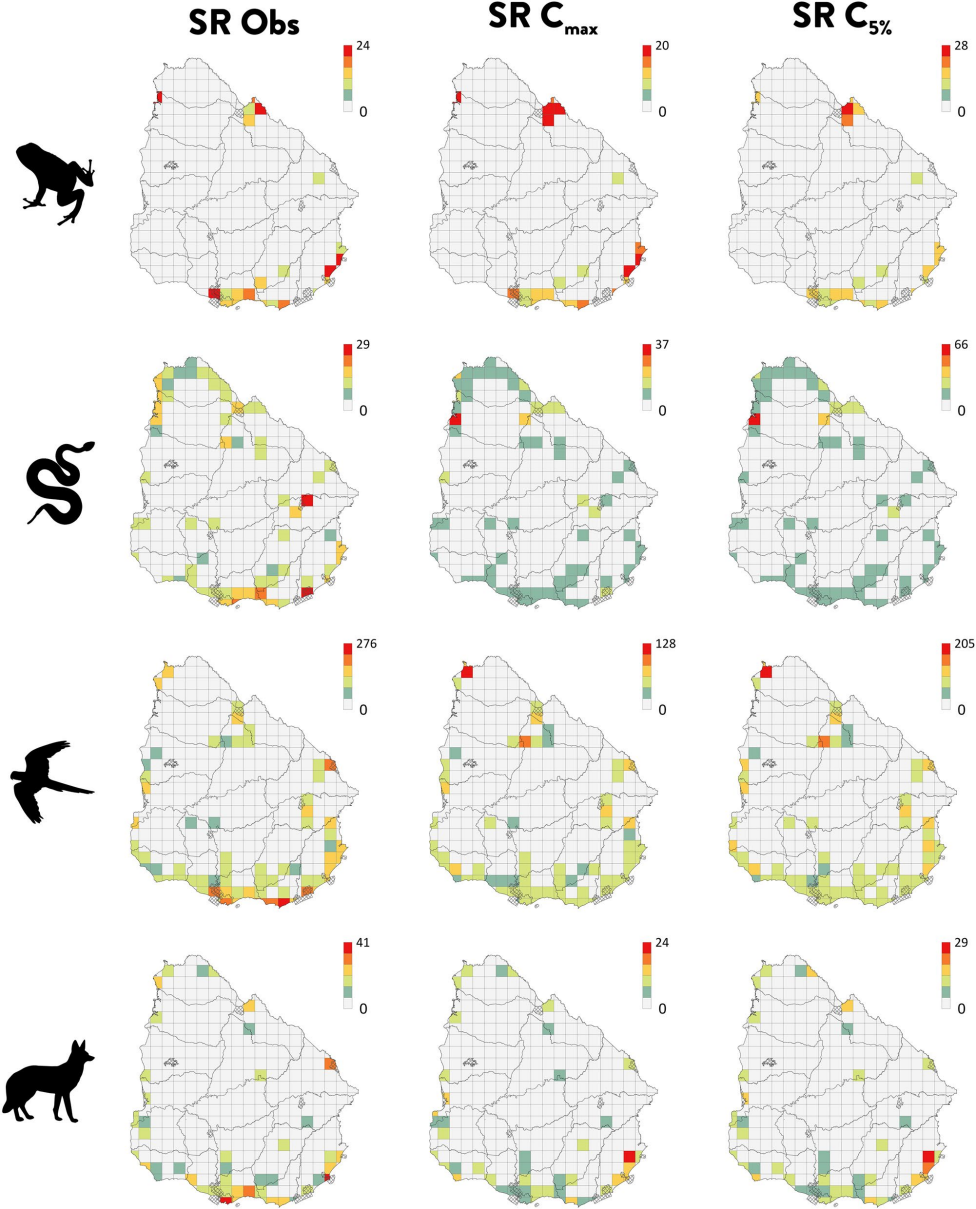
Spatial patterns of observed and estimated species-richness (Hill's number of order q = 0) for tetrapods in Uruguay. Observed species-richness, species-richness at C_max_ (minimum coverage of samples extrapolated to double the size of the reference sample) and at C_5%_ (5% percentile of sampling coverage at doubled sample sizes), for amphibians, reptiles, birds and mammals. Protected areas are shown overlapped. All maps in 25 × 25 km grid-cell resolution. Projection WGS1984. Maps generated using ArcGIS 10.6 (https://desktop.arcgis.com).

The number of grid-cells that were sufficiently sampled to be considered for the analyses varied across scales and taxa (see Supplementary Table S3 for specific numbers at each spatial resolution). Yet, for each taxonomic group, only between 7–21% and 34–62% of the grid-cells were included, for the 25 × 25 and 50 × 50 km grid-cell size, respectively. Amphibians were the group with the highest coverage levels (at C_max_ and C_5%_) regardless of the scale, while reptiles were the group with the lowest values of sampling coverage (Supplementary Table S3). The distribution patterns of the observed species-richness levels, across groups and scales, were usually not congruent with those of the species-richness standardised for sampling coverage at C_max_ and C_5%_ values (Fig. 3, Supplementary Fig. S5), though, the patterns of richness at C_max_ and at C_5%_ were generally consistent. For reptiles, birds and mammals, estimated species-richness was higher in northern areas, opposite to the peaks of observed richness seen in the coast (Fig. 3). For amphibians, maximum values of observed or estimated values of species-richness were more similarly distributed, occupying both northern and southern coastal regions (Fig. 3).

### Protected area network and hotspots of tetrapods

The network of protected areas (Supplementary Fig. S6) only partially encompasses hotspots in Uruguay (Supplementary Table S4). On average 56.7% of the richest grid-cells of observed tetrapod species-richness overlapped in part with a protected area (i.e., some extent of the hotspot was within a declared area), whilst overlapping decreased to 43.4% when considering richness standardised for sampling coverage at C_max_ and to 48.6% for richness estimated at C_5%_. Mammals species-richness peaks were better covered (50–66.7%) while reptiles’ hotspots were the poorest integrated (16.7–50%). In the case of hotspots of endemism, we saw that on average for all tetrapods 54.6% of the peaks were located within protected areas. For threatened species number we found that an average of 58.8% and 65.6.3% of the peaks were covered, considering global and national IUCN assessments respectively. Hotspots of threatened species proportion, however, did not overlap with protected areas, except for the amphibian’ group for which we found a 25% of overlapping.

### Areas of ‘ignorance’

**Figure 4:**
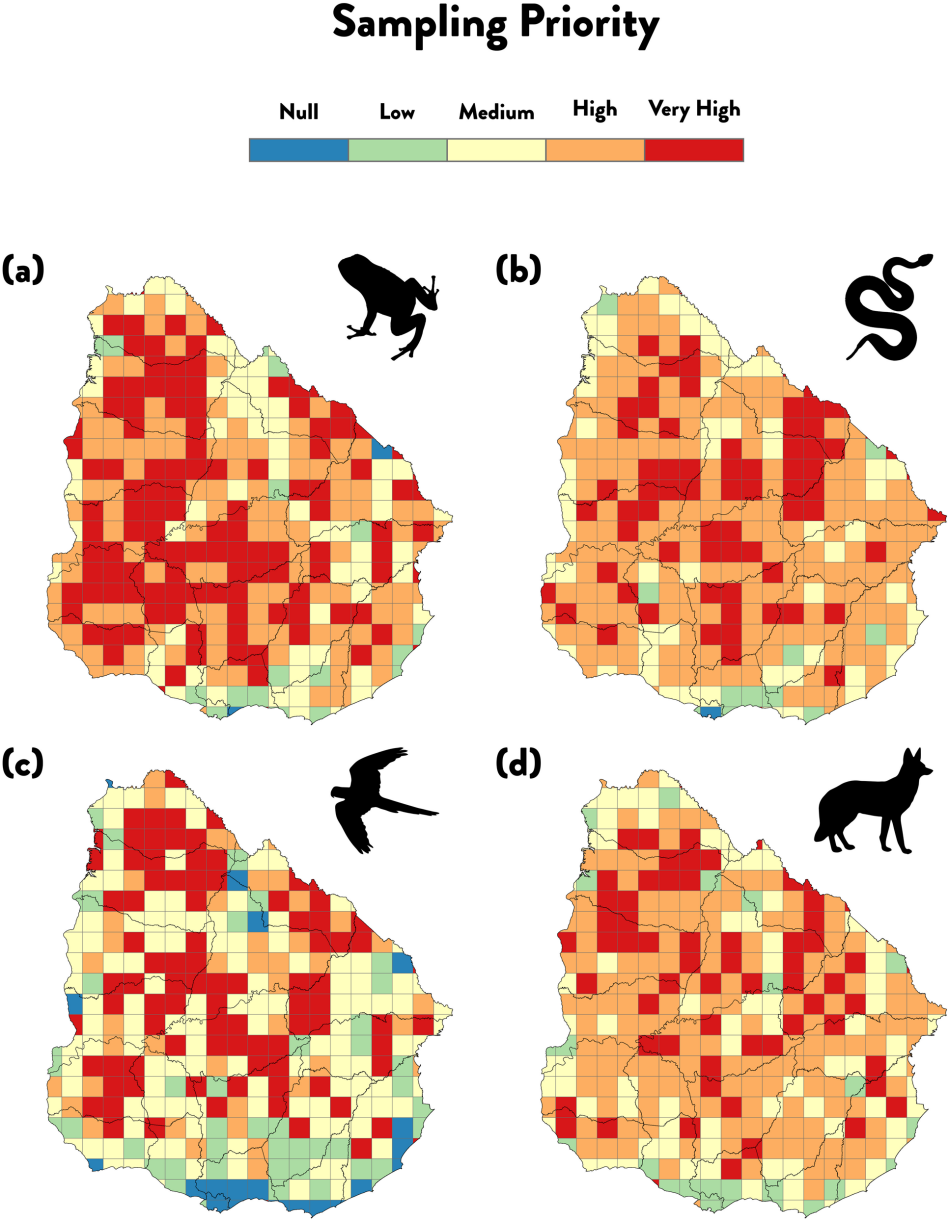
Areas for future sampling effort prioritisation. Priority categories for amphibians (**a**), reptiles (**b**), birds (**c**) and mammals (**d**) were calculated using species accumulation curves (SACs). Scale: Null (i.e., grid-cells where mean slope of the last 10% of SACs was lower or equal to 0.05), ‘Low’ (between 0.05 and 0.25), ‘Medium’ (between 0.25 and 1), ‘High’ (grid-cells where the sampling effort was so low that it was not possible to calculate SACs), and ‘Very High’ (i.e., grid-cells where no records were found). All maps in 25 × 25 km grid-cell resolution. Projection WGS1984. Maps generated using ArcGIS 10.6 (https://desktop.arcgis.com).

The spatial evenness in the distribution of the > 69,000 geographic records was low, and the levels of incompleteness per-area were considerably high (Fig. 4), with most of the territory (> 95.5%) identified as under-sampled (see SAC slope > 0.05; Supplementary Table S5; Supplementary Fig. S7). For amphibians, the data covered 61% of the territory (Table 1: grid-cell size 25 × 25 km), and yet, only two grid-cells can be considered as well sampled (SAC slope ≤ 0.5), covering only 0.3% of Uruguay’s area (Supplementary Fig. S7a). Birds, the group with the highest sampling effort, also had low levels of geographic coverage with 29.5% of the area unsampled (Table 1, Supplementary Fig. S7c), and with 20 grid-cells (4.5% of the national territory) considered as well-sampled. Reptiles and mammals showed the highest spatial coverage, with 77.2% and 79.5% of the total area covered, respectively (Table 1, Supplementary Fig. S7b,d). However, mammals did not present any well-sampled grid-cell and reptiles only 1, covering 0.2% of Uruguay’s area.

A major part of the Uruguayan territory was considered under the high and very-high sampling priority categories and in average for all tetrapods 67.5% of the area has been completely neglected (Fig. 4, Supplementary Table S5). These areas were mostly concentrated in the centre lowlands of the country.

## Discussion

Our study provides a detailed empirical case revealing the severe consequences that the lack of open-access biodiversity databases can have for the implementation of effective conservation actions directed to a country’s biodiversity management. By focusing on the tetrapod biodiversity of Uruguay—one of America’s most neglected countries in terms of availability of scientific data on its biodiversity^58^—our results show how the non-systematic (i.e., lacking a structured strategic approach for symmetric coverage of areas) and geographically concentrated sampling in only a few areas (at the expense of the majority of the country’s surface) have prevented the opportunities to identify areas of potential conservation and management priority. To address this issue, we have created the first open-access biodiversity initiative (Biodiversidata) in Uruguay, which reveals the distribution of different hotspots of biodiversity, and shows low levels of congruence among these measures regardless of the spatial scale or the IUCN assessment level used to calculate threatened species hotspots (i.e., national or global). Additionally, we identified well-surveyed sites, spatial gaps, and priority areas for future sampling efforts of amphibians, reptiles, birds and mammals in Uruguay. Thus, we believe that the novel evidence presented in our study will provide a critical scientific tool to effectively allocate resources for the exploration and monitoring of Uruguay’s biodiversity, and to ultimately enhance the efficiency in the process of evidence-based decision making towards conservation.

### Biodiversity hotspots: real or fabricated?

We found that number of species and endemism tend to concentrate around southern coastal cities. Studies performed at regional scales (e.g., sampling units > 2500 km^2^), have reported positive correlations between human population density and species-richness^60,61^. However, we cannot distinguish if the distribution of hotspots surrounding Uruguay’s major Atlantic-coast cities are a true pattern or an artefact product of sampling effort^62^, given that similar diversity levels could be found at other locations if sampling was as intense. By assessing true diversities (i.e., species-richness for a standardised sampling coverage), we found that the number of comparable areas is highly limited, yet those analysed tended not to exhibit the same distribution of species-richness peaks across scales and taxa. Thus, the question whether richness levels in the centre of the country are biologically (rather than artifactually) low, remains open, given the predominant knowledge gaps at these locations. Likewise, the high spatial correlation of species-richness for reptiles and amphibians, and of endemism for birds and mammals, cannot be disentangled from the effect of what could be essentially coordinated efforts of data collection linking these groups (e.g., herpetologist collectors). Importantly, incompleteness in the inventories may also correspond to existing knowledge that is not digital or accessible. In this sense, our analyses consider all the existing information that is possible to analyse, data that we rescued^63^ and made available.

### Spatial incongruence among hotspots of biodiversity

Discrepancies in the congruence of hotspots of biodiversity have previously been reported for other regions^37,64–67^. As the establishment of protected areas usually relies on the use of species-richness as a proxy of biodiversity^39^, the lack of congruence between different cross-taxon metrics debilitates the premise that a subset of taxa or features can be representative of biodiversity for conservation planning. Our findings suggest that the patterns we observe in Uruguay are strongly influenced by historical biases in sampling efforts that have dominated scientific practice. In this context, it is critical for future conservation assessments that we are able to quantify the spatial distribution of the different hotspots types, across taxa and at different scales of analysis^40^, to accurately recognise the limitations of the selection of reserve areas for the whole biodiversity protection and to monitor their effective representativeness.

### Towards effective conservation of biodiversity

The conservation prioritisation and planning strategies adopted by nations around the world are myriad. However, they all depend on comprehensive, high-resolution, up-to-date spatial information about species, ecosystems, and ecosystem services. The National System of Protected Areas of Uruguay was created in 2000, inaugurating the newest protected area system of Latin America. To prioritise which areas to include, the government collated a large biodiversity database (in grids of 660 km^2^—a very crude spatial resolution) that led to the decision of including specific areas despite the incompleteness of this nation-wide resource^68^—the Biodiversidata initiative aims to overcome this limitation^58^. In recent years, studies on the distribution of biodiversity have been performed using more complex quantitative methods^54^, yet, the data limitations have remained mostly the same. The bias that results from uneven sampling effort highly affects the estimation of richness^69,70^ and may lead to ineffective conservation prioritisation^71,72^, particularly in developing countries^73^. The efficiency of biodiversity conservation of the protected area system in Uruguay has not been tested. Precisely, in our study we observe that some areas need additional conservation attention to reach the most complete representation of the different tetrapod groups in the current network.

Uncertainty in the selection of suitable environments for conservation may lead to inadequate reserve selection and inappropriate habitat protection to higher extinction vulnerabilities^74^. Consequently, the allocation of investment for the study of neglected areas (particularly in countries such as Uruguay, where the dominant proportion of the country can be classed within this category) is likely to impact considerably on the efficiency of decisions and ultimately, on the expected outcomes^75^. Conservationists are often required to make decisions with incomplete and biased data, however, in order to improve and project better decision-making, there is an urgent need to focus in the knowledge gaps^76^. For instance, in the past 15 years, new species to science have been described and others have been recorded for the first time in the country expanding their distribution ranges, most of which are not considered on conservation prioritisation schemes. A range of new tetrapod species, including reptiles (*Contomastix charrua*^77^ and *Liolaemus gardeli*^78^), amphibians (e.g., *Rhinella achavali*^79^, *Melanophryniscus langonei*^80^ and *Odontophrynus maisuma*^81^), as well as first time species records including charismatic mammals (e.g., *Puma yagouaroundi*^82^ and *Alouatta caraya*^83^), amphibians (e.g., *Leptodactylus furnarius*^84^, *Boana albopunctata*^85^ and *Physalaemus cuvieri*^86^) and birds (e.g., *Piculus aurulentus*, *Myiarchus tyrannulus* and *Anthus nattereri*^87^, *Ramphastos toco*^88^ and *Tyrannus tyrannus*^89^).

### Future directions targeting knowledge gaps

Gaps in digital accessible information about the geographical distribution of species are a well-known and global issue^28,90^ that precludes from informing or monitoring the accomplishment of conservation targets across continents^76^. Open-access standardised datasets^91^ on species taxonomy, distribution, abundance, and evolutionary patterns remain largely unavailable in Uruguay, for all groups across the tree of life—this makes Uruguay one of America’s most neglected countries in this sense. Remarkably, our results reveal that for tetrapods, > 95.5% of the country’s land area remains insufficiently sampled. Thus, in the near future, biodiversity data mobilisation^92^ is amongst the greatest challenges the country will face^93,94^. Currently, the major scientific collections (i.e., Universidad de la República and the Museo Nacional de Historia Natural de Uruguay) are digitally inaccessible and, therefore, at latent risk of being lost^95^. Key efforts need to be made to support research institutions, researchers, policy makers and other stakeholders to digitise and store biodiversity data, and to guarantee its availability for evidence-based environmental planning and management^76^. Importantly, field research and data-sharing practices need to be encouraged. In this regard, our work provides a detailed roadmap of areas where to increase efforts for each tetrapod group. Lastly, as it is to many other non-western countries^96^, citizen science data (e.g., eBird, iNaturalist) has proven to bear a remarkable potential in documenting and monitoring biodiversity^97,98^, and therefore, the promotion of public engagement and knowledge democratization processes in countries like Uruguay can play an important role in channelling the needed scientific-culture change.

## Materials and methods

### Data

Geographic occurrence data of the tetrapods of Uruguay were collated from original sources collected by Biodiversidata expert members, from online databases and from the scientific literature (see details and protocols in Grattarola, et al.^58^). To avoid over-inflation of the data, all duplicates species per locality/year (i.e., same geographic coordinates) were removed. After this process of data filtering, the total number of records was 69,364 (Table 1), covering 664 tetrapod native species. This is the most geographically and taxonomically comprehensive database of Uruguay’s biodiversity that has been collected to date. The complete database is available at Grattarola, et al.^99^ and Grattarola, et al.^100^.

### Mapping biodiversity metrics and hotspots

We considered two diversity metrics to define biodiversity hotspots^19^ (number of species per area, or species-richness, and the proportion of species restricted to a particular area, or endemism); and two measures of species vulnerability (the proportion of threatened species relative to total species numbers, and the number of threatened species). We used the range-size-weighted species-richness (rswSR) as our measure of endemism, a parameter that considers the rarity/prevalence of the species over the study area. This enabled us to account for the predominance of species with restricted geographic distribution in the country, for simplicity we refer to it as ‘endemism’. The rswSR was calculated following Roll, et al.^32 ^Eq. (1),

1 $${\text{rswr}}_{{\text{i}}} = \sum_{{\text{j}}} {\text{q}}_{{{\text{ij}}}} ,$$

where q_ij_ is the fraction of the distribution of the species j in the cell i. Threatened species number was calculated counting the number of species listed as threatened and threatened species proportion as the fraction of species listed as threatened per grid-cell, following Böhm, et al.^18 ^Eq. (2),

2 $${\text{Prop}}_{{{\text{Threat}}}} = \left( {{\text{CR}} + {\text{EN}} + {\text{VU}}} \right){\text{/N}},$$

including critically endangered (CR), endangered (EN) and vulnerable (VU) categories by the total number of species (N). For both measures of threatened species we used the IUCN Red List of Threatened Species global assessment^101^ and the IUCN Red List national assessments for amphibians and reptiles^102^, and birds^103^. We only used the global assessment for mammals given there is no IUCN assessment for this group at the national level. Thus, threatened species analyses combining all tetrapods were done considering only global categories.

We defined hotspots as a measure of the spatial distribution of diversity/vulnerability metrics (i.e., species-richness, endemism or threatened species proportion and number), as a function of grid-cells rather than an arbitrary cut-off point (e.g., 2.5% of the richest areas). Therefore, we assumed that hotspots were the highest extremes of a gradient of continuous variation.

Analysing spatial patterns using different scales of observation can be useful when the size of the unit (grid-cell in this case) at which the spatial structure can be characterised is unknown^104^. Thus, we performed all analyses using three different sizes: 50 × 50, 25 × 25 and 12.5 × 12.5 km. Although all the analyses were made for the three different grid-cells sizes, we report results from the analyses with the 25 km grid-cell size (see Supplementary Fig. S2,S3 for analyses with the other two grid-cell sizes). All maps for each group separately (amphibians, birds, reptiles and mammals) and for all tetrapods combined were created using ArcGis 10.6. Sampling effort was evaluated as the number of records in each cell (after filtering for pseudo-replication) and species-richness as the number of species corresponding to those records.

### Hotspots congruence

We assessed the extent of congruence for each hotspot type (species-richness, endemism and threatened species proportion and number), within each group and across the tetrapod group, by calculating the number of overlapping grid-cells^37^. In cases where data are not randomly distributed, measuring metrics with small sampling units will increase the variance while using large sampling units will reduce the variability^104^, which therefore limits our capacity to determine the congruence among hotspots. For this reason, to analyse the extent of congruence between the biodiversity hotspots we varied both the size of the sampling unit (12.5 × 12.5, 25 × 25 and 50 × 50 km) and the criterion to define a hotspots (% of area/number of cells occupied by hotspots). First, grid-cells without records were removed. Then we sorted all the cells from high to low values of the corresponding metrics. Finally, at each definition criterion (from 0 to 100% overlapping over the total area by 0,5%), we computed the percentage of congruence as the number of matching grid-cells over the total number of unique cells. See the script with a working example in Grattarola^105^.

### Spatial correlations

We assessed the spatial association between: (1) sampling effort versus each hotspot type, (2) pair of hotspot types within each tetrapod group (e.g., amphibians’ species-richness versus amphibians’ endemism), and (3) pair of tetrapod classes within each hotspot type (e.g., reptiles’ endemism vs. birds’ endemism). Ecological data often have some degree of spatial structure^104^,—grid-cells can show a tendency to have similar values for a given variable with closely distributed grid-cells (i.e., spatial autocorrelation). Therefore, it is important to control whether spatial autocorrelation exists among grid-cells for each hotspot metric, and if so, estimate the ‘effective sample size’ given the dependency among the values^104^. Thus, to measure the association between the number of records and the biodiversity metrics per grid-cell we used a corrected Pearson’s correlation for spatial autocorrelation^104,106^ of the ‘SpatialPack’ R package^107^. Cells without records were eliminated from all correlations to remove double zeros.

### Comparisons of observed and estimated diversities

Although the question of whether patterns of species-richness are real (the recovered pattern represents the true distribution of biodiversity) or fabricated (the recovered pattern is an artefact of the distribution of sampling efforts) cannot be accurately assessed for severely under-sampled assemblages, we can still infer species-richness for a standardised coverage and make comparisons between observed and estimated diversity values^108,109^. Fair comparisons across multiple groups can be performed using coverage-based rarefaction and extrapolation sampling curves up to a maximum value of C_max_ (i.e., the level of coverage reached by the sample that attains the lowest coverage when all samples are extrapolated to double the reference sample size)^109^.

We defined the frequency of species incidence at two spatial resolutions: 25 × 25 and 50 × 50 km grid-cells (we did not use the 12.5 × 12.5 km resolution due to low numbers of available grid-cells with data for analyses). Each grid-cell was further divided into sub-grids of 1 × 1 km to create a species incidence dataset at the grid-cell level by counting the number of sub-gridded cells that contained occurrence records for individual species (see the script with a working example in Grattarola^105^). For interpolation (rarefaction) and extrapolation of species-richness (Hill's number of order *q* = 0) we used the R package ‘iNext’^110^. To obtain reliable estimates, grid-cells with few occurrence records were excluded from analyses^111^: whenever the number of species observed in the grid was less than six, the number of sub-gridded cells with at least one incidence was less than six, and the total number of species incidences was equal to the number of unique species (species that are each detected in only one sub grid-cell). To compare among grid-cells in terms of sampling coverage, we estimated species-richness for each tetrapod group at C_max_^109^ and at 5% percentile (C_5%_) of sampling coverage at doubled sample sizes^111^. To determine C_max_, each sample within the grid unit was first extrapolated to double the reference sample size, then C_max_ was calculated as the minimum among the coverage values obtained from those extrapolated samples. See Supplementary Table 3 for specific values at each group and spatial resolution.

### Congruence between protected areas and hotspots of biodiversity

To determine whether the location of existing protected areas in Uruguay (as established by the current National System of Protected Areas of Uruguay) covers hotspots of species-richness, endemism and threatened species, all maps were overlapped with the 16 currently operating protected areas (see Supplementary Figure S4 for a map showing: the network of protected areas, areas under assessment for potential consideration as protected areas, and areas for which a proposal for consideration has been prepared). Congruence for each hotspot type was then calculated as the proportion of the 2.5% of the richest grid-cells, removing empty grid-cells, that were at least partially covered by a protected area (i.e., some amount of the area was within a hotspots).

### Identification of ‘areas of ignorance’

As a critical first step to interrogate historically-established public policies and efforts about the sampling of Uruguay’s biodiversity, we quantified the levels of inventory incompleteness for each group per area by using curvilinearity of smoothed species accumulation curves (SACs)^112,113^. This method assumes that SACs of poorly sampled grid-cells tend towards a straight line, while those of better sampled ones have a higher degree of curvature^114^. Smoothed SACs were calculated with the method ‘exact’ of the function ‘specaccum’ in the vegan R package^115^. As a proxy for inventory incompleteness we calculated the degree of curvilinearity as the mean slope of the last 10% of SACs^112^. Steep slopes (values close to one) reflected high levels of incompleteness, whereas shallow slopes (values close to zero) indicated saturation in the sampling and thus low levels of incompleteness. We considered grids with slope values > 0.05 to be under-sampled and those with slope values ≤ 0.05 to be well sampled. The R scripts used for these analyses can be found at Grattarola^105^.

Finally, with the aim to outline a plan to suggest where future sampling efforts should be allocated across the territory of Uruguay (e.g., for funding considerations), we generated a map of ‘priority areas of sampling’ for each tetrapod group. Priority levels were established considering the levels of inventory incompleteness. The scale ranged from: Null (i.e., grid-cells where mean slope of the last 10% of SACs was lower or equal to 0.05), ‘Low’ (between 0.05 and 0.25), ‘Medium’ (between 0.25 and 1), ‘High’ (grid-cells where the sampling effort was so low that it was not possible to calculate SACs), and ‘Very High’ (i.e., grid-cells where no records were found).

## Supplementary Information

Supplementary Information 1.

Supplementary Information 2.

## Data Availability

All the primary data on species occurrence is available at Zenodo (http://doi.org/10.5281/zenodo.3685897). The data provided by the members of Biodiversidata can also be accessed via GBIF.org (https://doi.org/10.15468/ozcrpu).The scripts to perform analyses of hotspots congruence, identification of areas of ‘ignorance’ and spatial correlations can be found in GitHub (https://github.com/bienflorencia/biodiversity_hotspots).
