# Supplementary material for: Multiple forms of hotspots of tetrapod biodiversity and the challenges of open-access data scarcity
Source: Sci Rep. 2020 Dec 16;10:22045. doi: 10.1038/s41598-020-79074-8 (PMC7745038; doi:10.1038/s41598-020-79074-8)
Supplement: Supplementary file 1 — Supplementary Information 1. [file 41598_2020_79074_MOESM1_ESM.docx]

**Supplementary Figures**

**Multiple forms of hotspots of tetrapod biodiversity and the challenges of open-access data scarcity**

Florencia Grattarola, Juan A. Martínez-Lanfranco, Germán Botto, Daniel E. Naya, Raúl Maneyro, Patricia Mai, Daniel Hernández, Gabriel Laufer, Lucía Ziegler, Enrique M. González, Inés da Rosa, Noelia Gobel, Andrés González, Javier González, Ana L. Rodales & Daniel Pincheira-Donoso.

**Includes Supplementary Figures S1-S7**


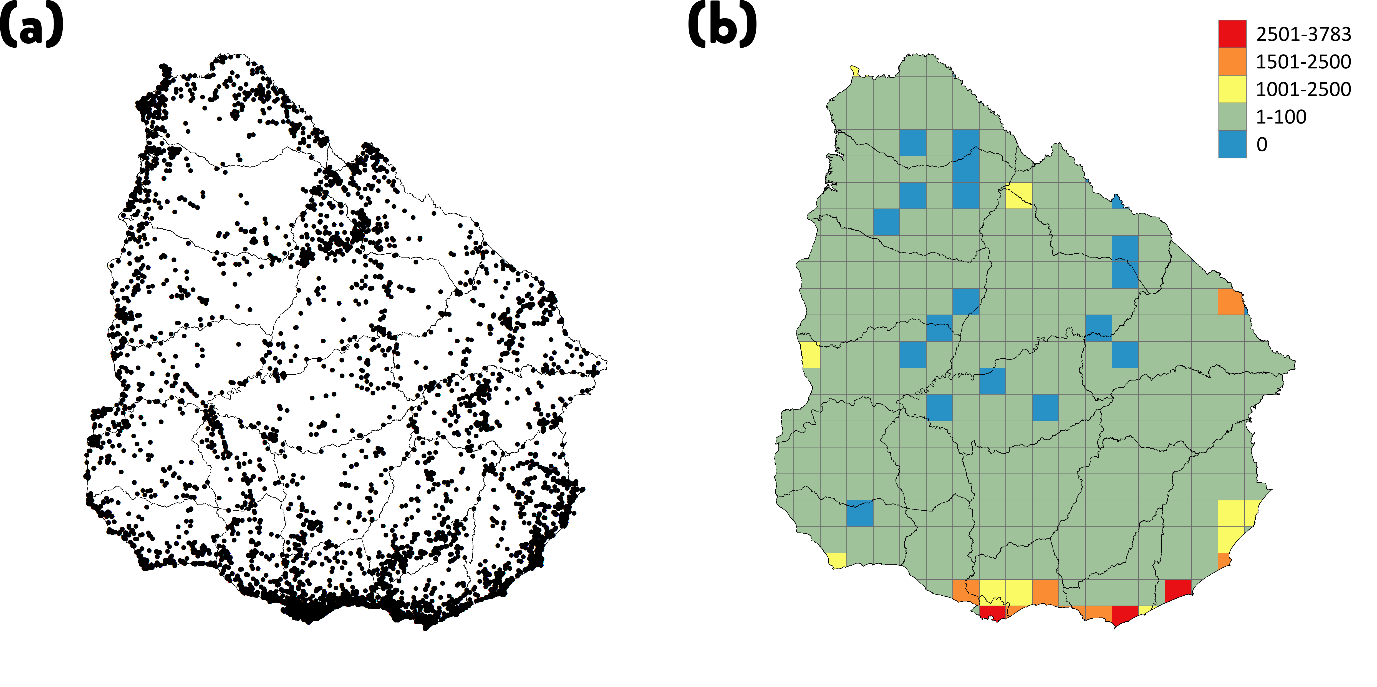


**Supplementary Figure S1.** Spatial distribution of the 69,364 occurrence records of tetrapod species in Uruguay. Point density (a) and sampling effort map in 25 × 25 km grid-cell resolution (b). Projection WGS1984. Maps generated using ArcGIS 10.6 (https://desktop.arcgis.com).


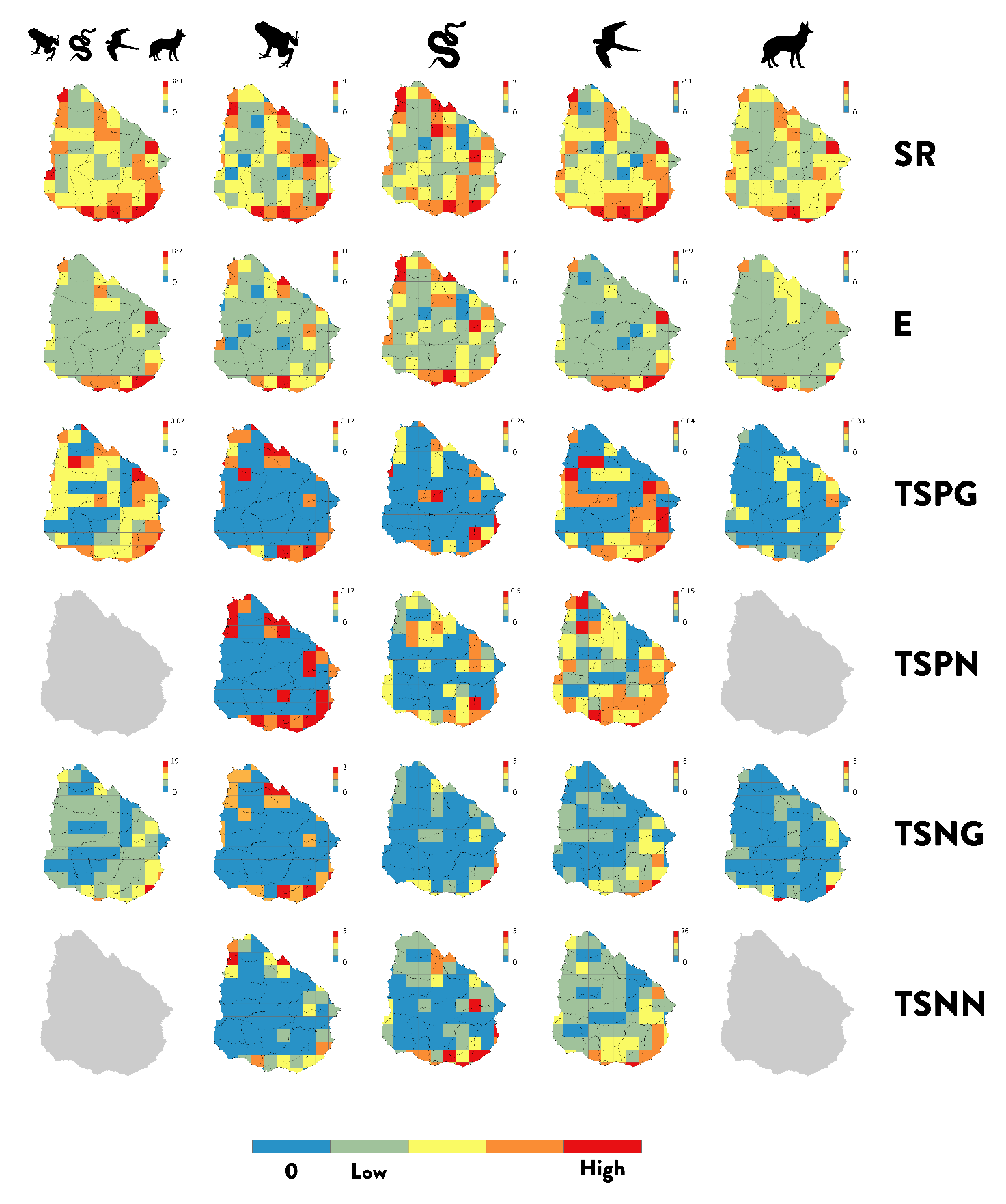


**Supplementary Figure S2.** Spatial distribution patterns of hotspots of tetrapod species in Uruguay. Hotspots metrics for all tetrapods, amphibians, reptiles, birds and mammals (from left to right), of species-richness (SR) (top row), endemism (E) (second from the top row), threatened species proportion using the global IUCN assessment (TSPG) (third row) or national IUCN assessment (TSPN) (forth row), and threatened species number using the global IUCN assessment (TSNG) (fifth row) or national IUCN assessment (TSNN) (bottom row). Blue cells lack any observations. Because of lack of a national mammals’ threat assessment, values for mammals and tetrapods at national level could not be computed (grey maps are shown). Scale bar values differ between panels depending on the hotspot’s metric. All maps in 50 × 50 km grid-cell resolution. Projection WGS1984. Maps generated using ArcGIS 10.6 (https://desktop.arcgis.com).


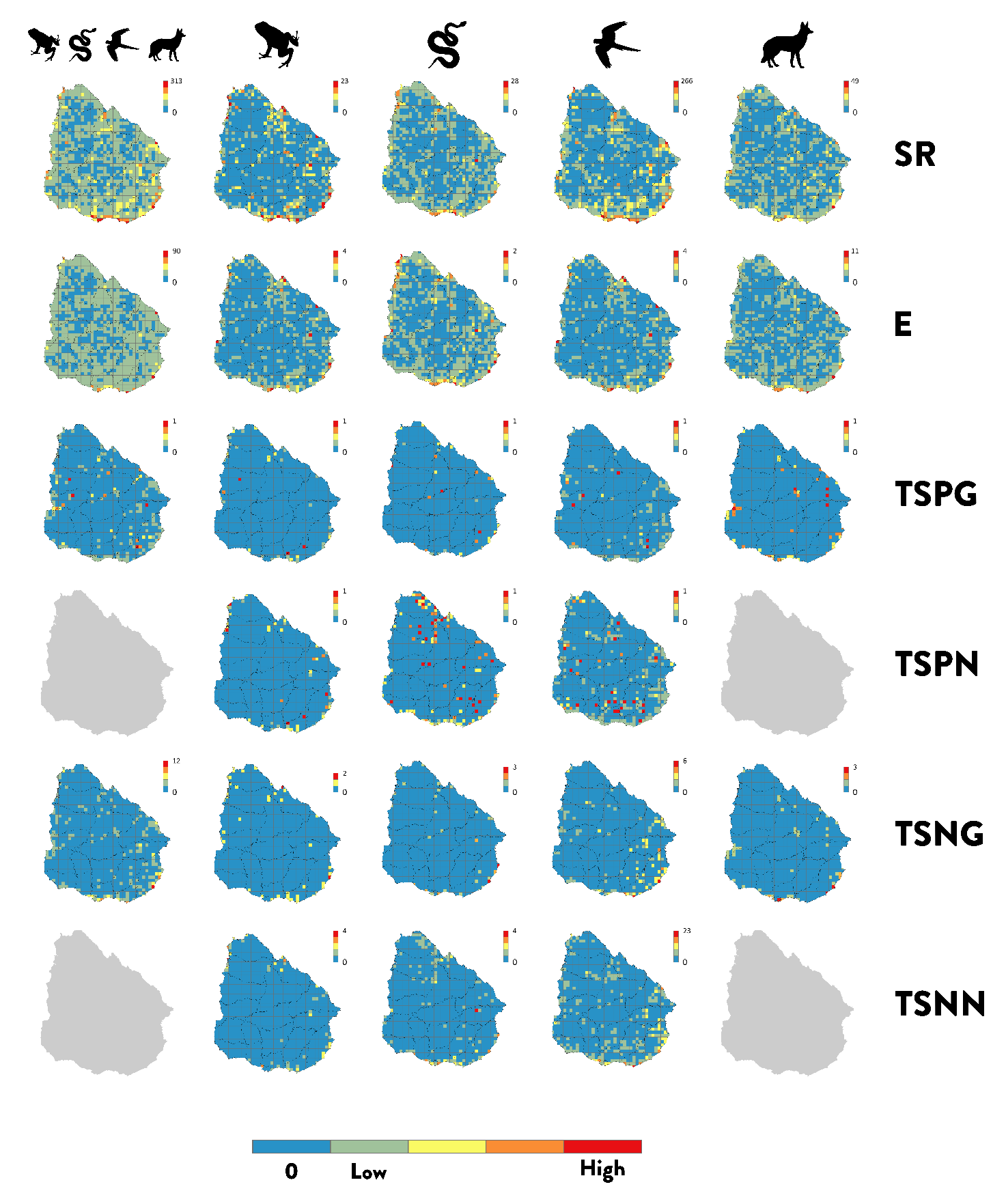


**Supplementary Figure S3.** Spatial distribution patterns of hotspots of tetrapod species in Uruguay. Hotspots metrics for all tetrapods, amphibians, reptiles, birds and mammals (from left to right), of species-richness (SR) (top row), endemism (E) (second from the top row), threatened species proportion using the global IUCN assessment (TSPG) (third row) or national IUCN assessment (TSPN) (forth row), and threatened species number using the global IUCN assessment (TSNG) (fifth row) or national IUCN assessment (TSNN) (bottom row). Blue cells lack any observations. Because of lack of a national mammals’ threat assessment, values for mammals and tetrapods at national level could not be computed (grey maps are shown). Scale bar values differ between panels depending on the hotspot’s metric. All maps in 12.5 × 12.5 km grid-cell resolution. Projection WGS1984. Maps generated using ArcGIS 10.6 (https://desktop.arcgis.com).


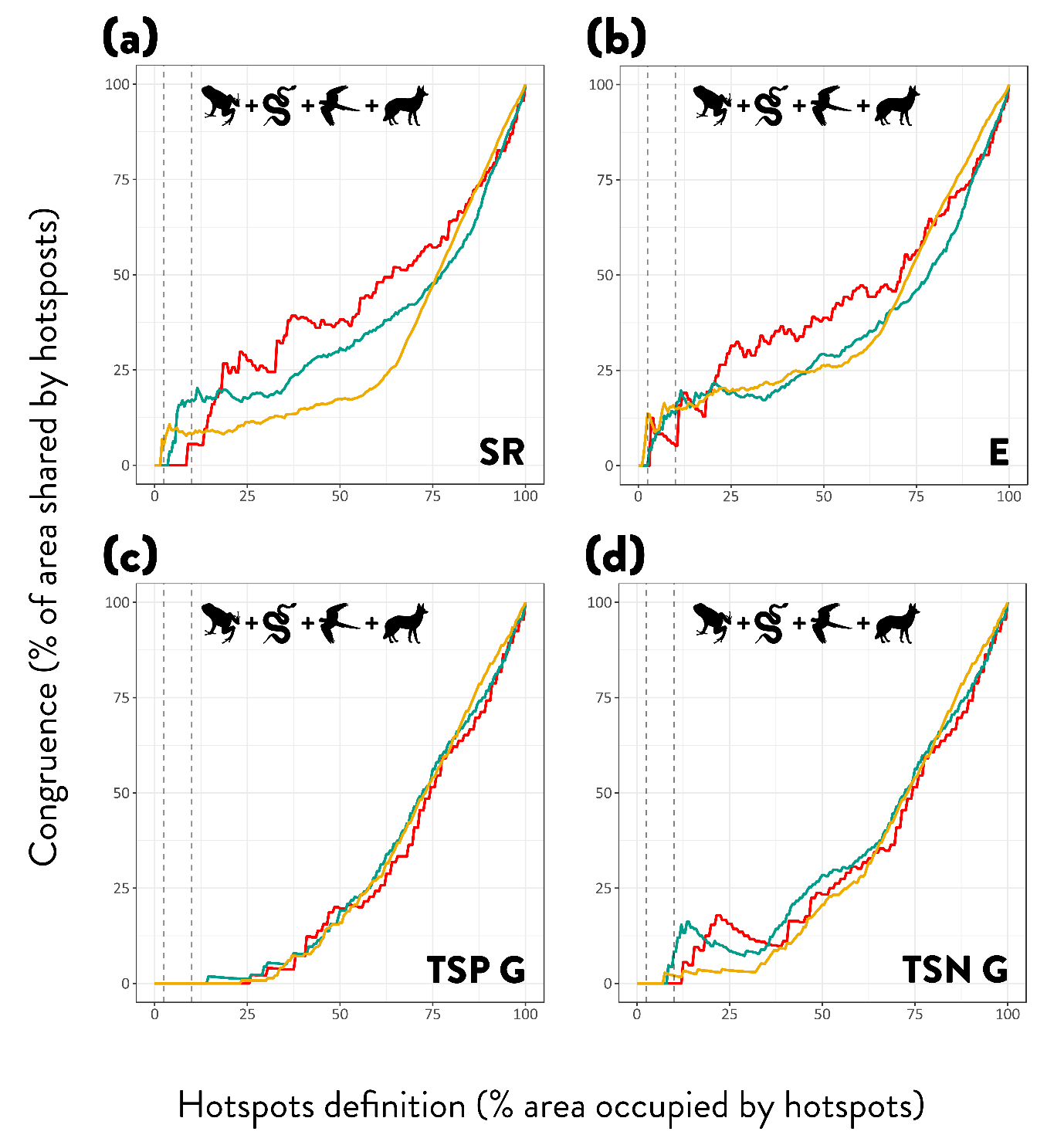


**Supplementary Figure S4.** Extent of hotspots’ spatial congruence across all tetrapods. Species richness (a), endemism (b), threatened species proportion using global IUCN status (c) and threatened species number using global IUCN status (d). Metrics measured with three different grid-cell sizes 50x50km (red line), 25x25km (green line) and 12.5x12.5km (yellow line). Congruence is the number of cells that represent hotspots for all four tetrapods’ group and is measured as the percentage of shared grid-cells over the percentage of land covered by hotspots according to a varying definition from 0 to 100% of the total grid-cells. Vertical dashed lines show 2.5% and 10% hotspot definition criterion.

**
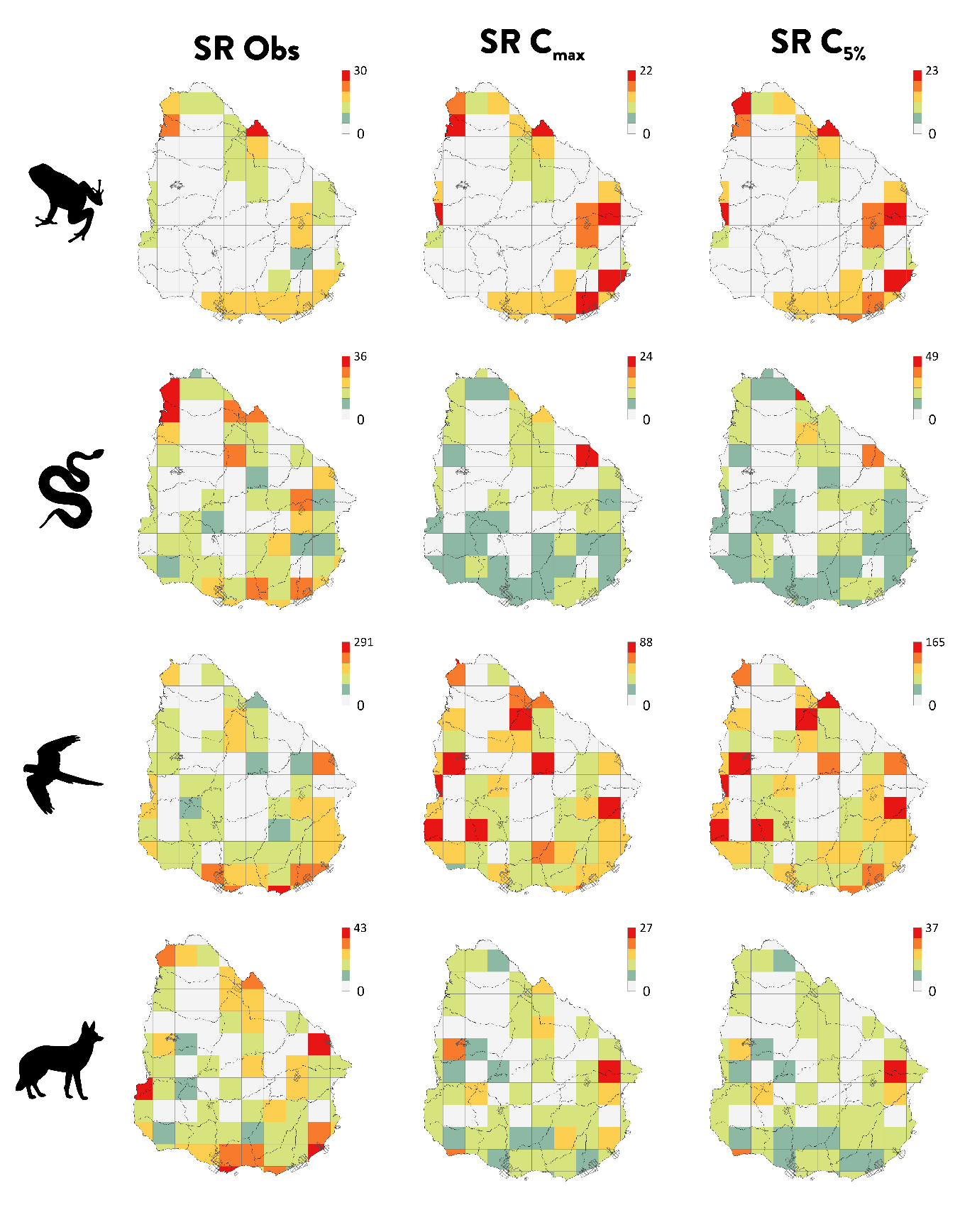
**

**Supplementary Figure S5.** Spatial patterns of observed and estimated species-richness (Hill's number of order q = 0) for tetrapods in Uruguay. Observed species richness, species richness at C_max_ (minimum coverage of samples extrapolated to double the size of the reference sample) and at C_5%_ (5% percentile of sampling coverage at doubled sample sizes), for amphibians, reptiles, birds and mammals. Protected areas are shown overlapped. All maps in 50 × 50 km grid-cell resolution. Projection WGS1984. Maps generated using ArcGIS 10.6 (https://desktop.arcgis.com).

**
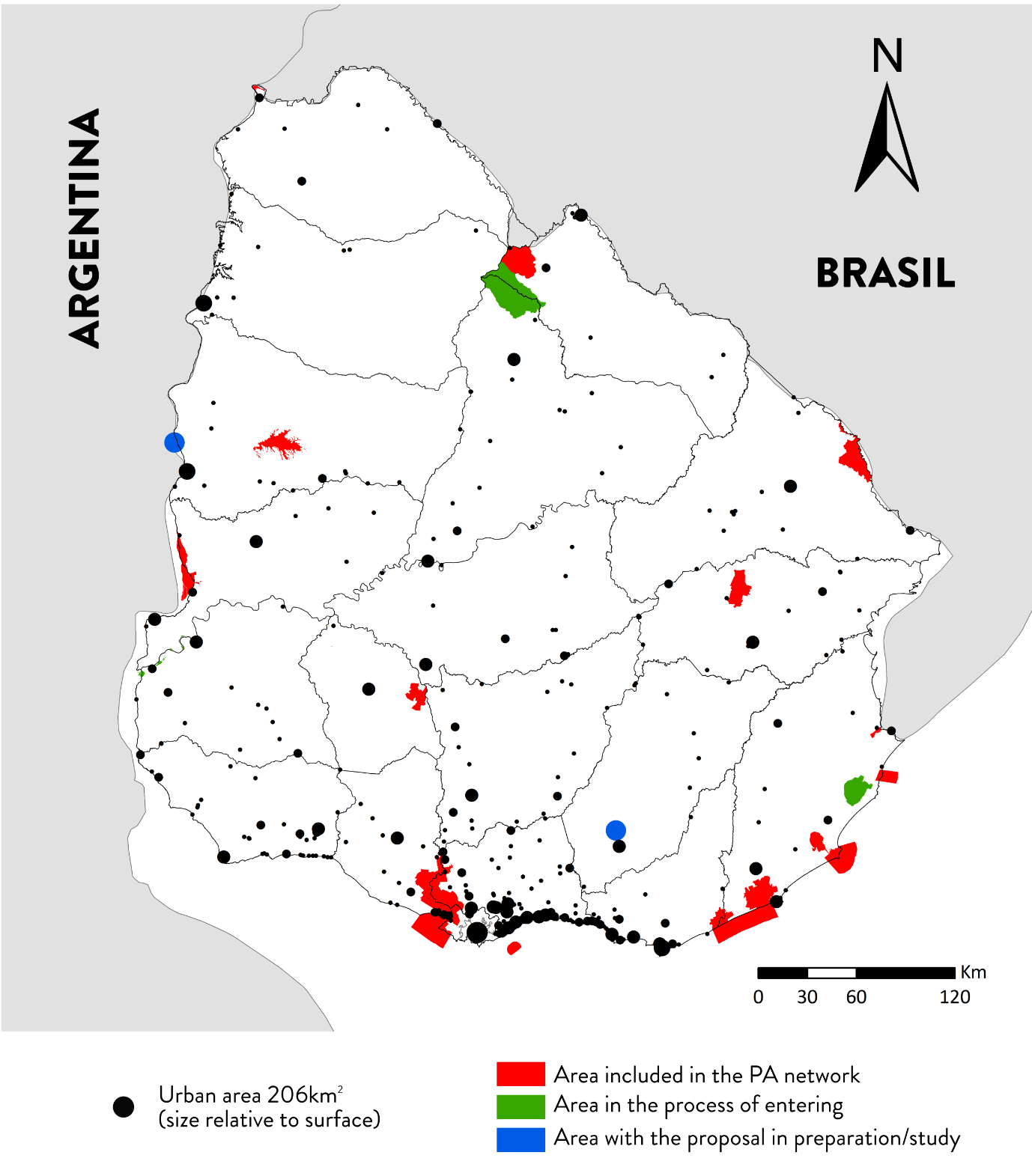
**

**Supplementary Figure S6.** Network of protected areas of Uruguay. In red protected areas included in the system, in green areas under assessment for potential consideration as protected areas (in process), and in blue areas for which a proposal for consideration has been prepared. Urban areas are shown as black dots with size relative to surface in km^2^. Map generated using ArcGIS 10.6 (https://desktop.arcgis.com).


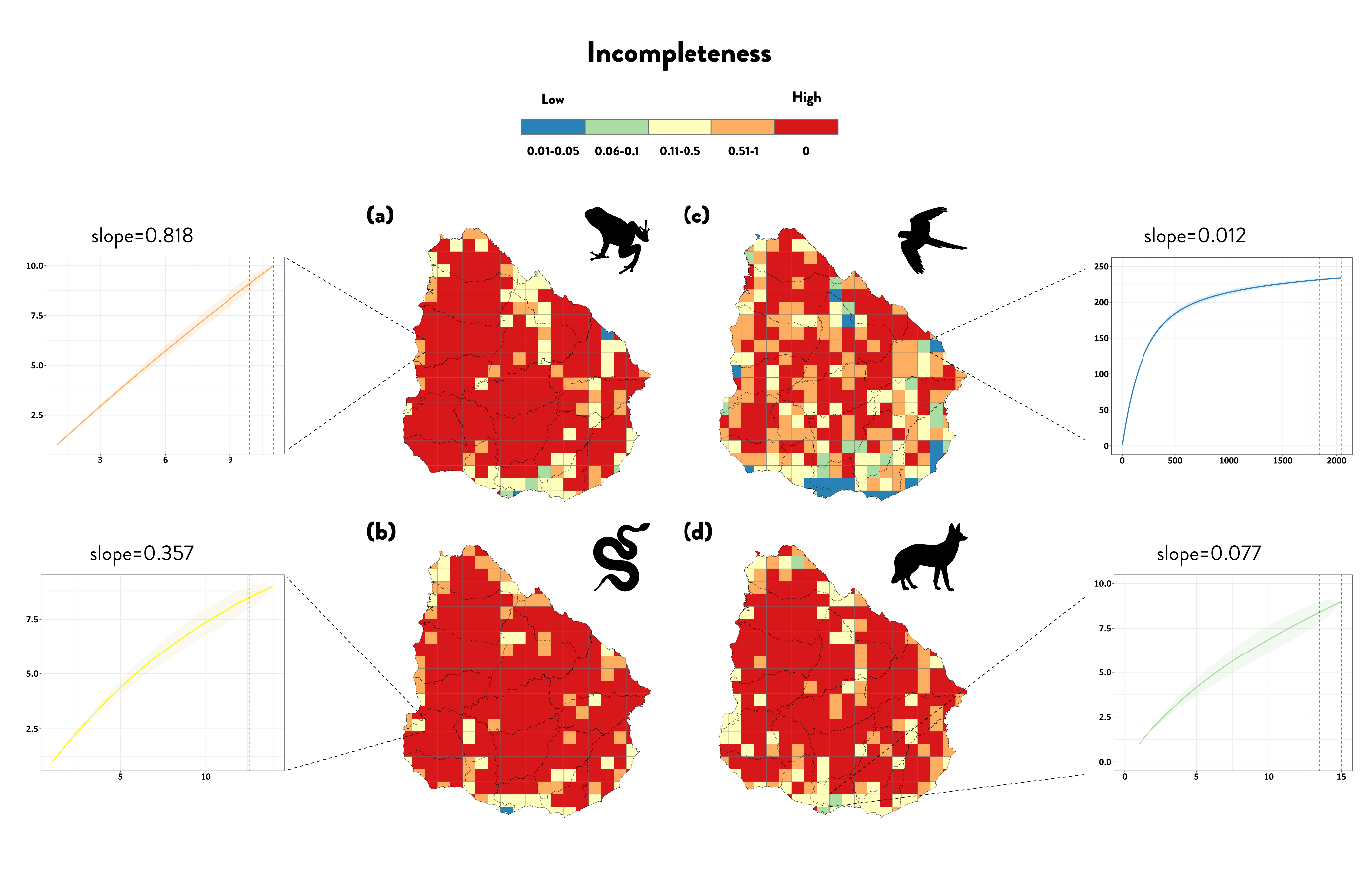


**Supplementary Figure S7.** Inventory incompleteness of tetrapods in Uruguay. Maps of species accumulation curves (SACs) with different curvilinearity, for amphibians (a), reptiles (b), birds (c) and mammals (d). Scale bar represent mean slope of the last 10% of SACs values; red areas represent areas that were not analysed due to null or low number of records to calculate SACs (high levels of inventory incompleteness), blue grid-cells are those with slope values ≤ 0.05 (well-sampled grids), green (slope between 0.06 and 0.1), yellow (slope between 0.11 and 0.5), orange (slope values > 0.51 and 1). Different degrees of curvilinearity are shown in four exemplary grid-cells for amphibians (a), reptiles (b), birds (c) and mammals (d). The portion between the vertical dashed lines indicates the last 10% of the species accumulation curves. All maps in an equal area, 25x25 km grid-cell resolution. Projection WGS1984 UTM zone 21S. Maps generated using ArcGIS 10.6 (https://desktop.arcgis.com).
