# Supplementary material for: Multiple forms of hotspots of tetrapod biodiversity and the challenges of open-access data scarcity
Source: Sci Rep. 2020 Dec 16;10:22045. doi: 10.1038/s41598-020-79074-8 (PMC7745038; doi:10.1038/s41598-020-79074-8)
Supplement: Supplementary file 2 — Supplementary Information 2. [file 41598_2020_79074_MOESM2_ESM.docx]

**Supplementary Tables**

**Multiple forms of hotspots of tetrapod biodiversity and the challenges of open-access data scarcity**

Florencia Grattarola, Juan A. Martínez-Lanfranco, Germán Botto, Daniel E. Naya, Raúl Maneyro, Patricia Mai, Daniel Hernández, Gabriel Laufer, Lucía Ziegler, Enrique M. González, Inés da Rosa, Noelia Gobel, Andrés González, Javier González, Ana L. Rodales & Daniel Pincheira-Donoso.

**Includes Supplementary Tables S1-S5**

**Supplementary Table S1.** Spatial correlations of number of records and biodiversity metrics (species richness, endemism, threatened species proportion global and national, and threatened species numbers global and national) for all tetrapods and each separate group, using Pearson’s correlation corrected for spatial autocorrelation. The correlations were performed with values per grid-cell at a 25km grid-cell resolution. Correlation coefficients (*r*), p-values and estimated degrees of freedom corrected for the spatial process (*edf*) are shown. Significance is shown in boldface.

|  | **Tetrapods** | | |  | **Amphibians** | | |  | **Reptilies** | | |  | **Birds** | | |  | **Mammals** | | |
| --- | --- | --- | --- | --- | --- | --- | --- | --- | --- | --- | --- | --- | --- | --- | --- | --- | --- | --- | --- |
|  | *r* | *p value* | *edf* |  | *r* | *p value* | *edf* |  | *r* | *p value* | *edf* |  | *r* | *p value* | *edf* |  | *r* | *p value* | *edf* |
| **Number of Records vs.** |  |  |  |  |  |  |  |  |  |  |  |  |  |  |  |  |  |  |  |
| Species Richness | 0.826 | ***<0.001*** | 117.2 |  | 0.830 | ***<0.001*** | 92.4 |  | 0.907 | ***<0.001*** | 133.6 |  | 0.800 | ***<0.001*** | 105.5 |  | 0.892 | ***<0.001*** | 165.7 |
| Endemism | 0.930 | ***<0.001*** | 145.7 |  | 0.865 | ***<0.001*** | 104.7 |  | 0.819 | ***<0.001*** | 126.8 |  | 0.915 | ***<0.001*** | 128.2 |  | 0.915 | ***<0.001*** | 171.8 |
| Threatened Species Number Global | 0.732 | ***<0.001*** | 119.9 |  | 0.597 | ***<0.001*** | 87.5 |  | 0.540 | ***<0.001*** | 184.6 |  | 0.644 | ***<0.001*** | 88.6 |  | 0.664 | ***<0.001*** | 182.6 |
| Threatened Species Proportion Global | 0.021 | 0.741 | 258.6 |  | 0.058 | 0.448 | 171.1 |  | 0.041 | 0.522 | 241.6 |  | 0.035 | 0.597 | 224.6 |  | 0.077 | 0.217 | 255.1 |
| Threatened Species Number National | - | - | - |  | 0.636 | ***<0.001*** | 88.0 |  | 0.633 | ***<0.001*** | 164.8 |  | 0.851 | ***<0.001*** | 90.1 |  | - | - |  |
| Threatened Species Proportion National | - | - | - |  | 0.119 | 0.192 | 120.6 |  | 0.023 | 0.750 | 195.5 |  | 0.000 | 0.995 | 225.4 |  | - | - |  |
|  |  |  |  |  |  |  |  |  |  |  |  |  |  |  |  |  |  |  |  |
| **Species Richness vs.** |  |  |  |  |  |  |  |  |  |  |  |  |  |  |  |  |  |  |  |
| Endemism | 0.743 | ***<0.001*** | 118.3 |  | 0.755 | ***<0.001*** | 93.0 |  | 0.802 | ***<0.001*** | 125.8 |  | 0.706 | ***<0.001*** | 114.8 |  | 0.768 | ***<0.001*** | 178.8 |
| Threatened Species Number Global | 0.714 | ***<0.001*** | 103.1 |  | 0.624 | ***<0.001*** | 65.3 |  | 0.521 | ***<0.001*** | 190.6 |  | 0.626 | ***<0.001*** | 90.4 |  | 0.558 | ***<0.001*** | 204.5 |
| Threatened Species Proportion Global | -0.028 | 0.659 | 251.7 |  | 0.041 | 0.598 | 166.4 |  | 0.018 | 0.771 | 256.5 |  | 0.003 | 0.963 | 234.9 |  | 0.055 | 0.382 | 256.7 |
| Threatened Species Number National | - | - | - |  | 0.664 | ***<0.001*** | 65.3 |  | 0.63 | ***<0.001*** | 172.7 |  | 0.793 | ***<0.001*** | 79.8 |  | - | - | - |
| Threatened Species Proportion National | - | - | - |  | 0.096 | 0.368 | 87.2 |  | 0.005 | 0.944 | 205.8 |  | -0.108 | 0.113 | 215.6 |  | - | - | - |
|  |  |  |  |  |  |  |  |  |  |  |  |  |  |  |  |  |  |  |  |
| **Endemism vs.** |  |  |  |  |  |  |  |  |  |  |  |  |  |  |  |  |  |  |  |
| Threatened Species Number Global | 0.712 | ***<0.001*** | 127.6 |  | 0.707 | ***<0.001*** | 82.6 |  | 0.681 | ***<0.001*** | 158.9 |  | 0.641 | ***<0.001*** | 118.7 |  | 0.729 | ***<0.001*** | 188.1 |
| Threatened Species Proportion Global | 0.035 | 0.574 | 266 |  | 0.12 | 0.116 | 170.8 |  | 0.124 | 0.054 | 239 |  | 0.044 | 0.503 | 230.2 |  | 0.133 | ***0.0343*** | 252.4 |
| Threatened Species Number National | - | - | - |  | 0.788 | ***<0.001*** | 81.5 |  | 0.613 | ***<0.001*** | 238.1 |  | 0.828 | ***<0.001*** | 98.2 |  | - | - | - |
| Threatened Species Proportion National | - | - | - |  | 0.22 | **0.020** | 109.3 |  | 0.087 | 0.147 | 274.7 |  | 0.021 | 0.756 | 222.0 |  | - | - | - |

**Supplementary Table S2.** Spatial correlations of biodiversity metrics between tetrapod groups (amphibians, reptiles, birds and mammals), using Pearson’s correlation corrected for spatial autocorrelation. The correlations were performed with values per grid-cell at a 25km grid-cell resolution. The correlations were performed with values per grid-cell at a 25km grid-cell resolution. Correlation coefficients (*r*), p-values and estimated degrees of freedom corrected for the spatial process (*edf*) are shown.

|  | **Species Richness** | | |  | **Endemism** | | |  | **Threatened Species Number Global** | | |  | **Threatened Species Proportion Global** | | |  | **Threatened Species Number National** | | |  | **Threatened Species Proportion National** | | |
| --- | --- | --- | --- | --- | --- | --- | --- | --- | --- | --- | --- | --- | --- | --- | --- | --- | --- | --- | --- | --- | --- | --- | --- |
|  | *r* | *p value* | *edf* |  | *r* | *p value* | *edf* |  | *r* | *p value* | *edf* |  | *r* | *p value* | *edf* |  | *r* | *p value* | *edf* |  | *r* | *p value* | *edf* |
| **Amphibians vs.** |  |  |  |  |  |  |  |  |  |  |  |  |  |  |  |  |  |  |  |  |  |  |  |
| Reptiles | 0.748 | ***<0.001*** | 119.3 |  | 0.656 | ***<0.001*** | 130.4 |  | 0.595 | ***<0.001*** | 143 |  | 0.022 | 0.697 | 325.3 |  | 0.467 | ***<0.001*** | 225 |  | 0.041 | 0.435 | 359.7 |
| Birds | 0.514 | ***<0.001*** | 120 |  | 0.36 | ***<0.001*** | 180.8 |  | 0.339 | ***<0.001*** | 205.2 |  | 0.069 | 0.222 | 317.3 |  | 0.478 | ***<0.001*** | 95.1 |  | 0.068 | 0.273 | 262.4 |
| Mammals | 0.672 | ***<0.001*** | 136.8 |  | 0.513 | ***<0.001*** | 222 |  | 0.348 | ***<0.001*** | 362.8 |  | 0.001 | 0.988 | 330.5 |  | - | - | - |  | - | - | - |
| **Reptiles vs.** |  |  |  |  |  |  |  |  |  |  |  |  |  |  |  |  |  |  |  |  |  |  |  |
| Birds | 0.531 | ***<0.001*** | 128.7 |  | 0.443 | ***<0.001*** | 130.1 |  | 0.369 | ***<0.001*** | 195.6 |  | 0.011 | 0.839 | 329.2 |  | 0.45 | ***<0.001*** | 198 |  | -0.017 | 0.768 | 305.1 |
| Mammals | 0.669 | ***<0.001*** | 144.8 |  | 0.598 | ***<0.001*** | 179.5 |  | 0.547 | ***<0.001*** | 268.1 |  | 0.029 | 0.602 | 324 |  | - | - | - |  | - | - | - |
| **Birds vs.** |  |  |  |  |  |  |  |  |  |  |  |  |  |  |  |  |  |  |  |  |  |  |  |
| Mammals | 0.587 | ***<0.001*** | 123 |  | 0.697 | ***<0.001*** | 195.8 |  | 0.416 | ***<0.001*** | 171.7 |  | -0.005 | 0.934 | 327.6 |  | - | - | - |  | - | - | - |

**Supplementary Table S3.** Numbers of analysed grid-cells for comparing true diversities for each tetrapod class and at each spatial resolution. For each group: number of analysed grid-cells (i.e.: sufficiently sampled to perform analyses), percentage of grid-cells excluded (%), value of C_max_ (i.e., minimum coverage of samples extrapolated to double the size of the reference sample) and value of C_5%_ (5% percentile of sampling coverage at doubled sample sizes).

|  | **Grids analysed** | | **% of grids excluded** | | **C_max_** | | **C_5%_** | |
| --- | --- | --- | --- | --- | --- | --- | --- | --- |
|  | **25x25** | **50x50** | **25x25** | **50x50** | **25x25** | **50x50** | **25x25** | **50x50** |
| Amphibians | 22 | 32 | 93.3 | 65.6 | 0.696 | 0.808 | 0.796 | 0.817 |
| Reptiles | 65 | 56 | 80.1 | 39.8 | 0.27 | 0.297 | 0.394 | 0.541 |
| Birds | 69 | 57 | 78.8 | 38.7 | 0.388 | 0.398 | 0.628 | 0.71 |
| Mammals | 46 | 58 | 85.9 | 37.6 | 0.312 | 0.359 | 0.388 | 0.447 |

**Supplementary Table S4.** Congruence of protected areas and hotspots of biodiversity.

For each hotspot type and tetrapod class congruence was calculated as the proportion of the 2.5% of the richest grid-cells that were at least partially covered by a protected area (i.e. some amount of the area was within a hotspots). Analyses were performed at the grid-cell size of 25x25 km.

| **Amphibians** |  | **Reptiles** |  | **Birds** |  | **Mammals** |  |
| --- | --- | --- | --- | --- | --- | --- | --- |
|  | **Overlap with PA (%)** |  | **Overlap with PA (%)** |  | **Overlap with PA (%)** |  | **Overlap with PA (%)** |
| **Species Richness** | 50 |  | 50 |  | 60 |  | 66.7 |
| **Species richness at C_max_** | 50 |  | 16.7 |  | 40 |  | 66.7 |
| **Species richness at C_5%_** | 75 |  | 16.7 |  | 20 |  | 50 |
| **Endemism** | 25 |  | 66.7 |  | 60 |  | 66.7 |
| **Threatened species number (global)** | 25 |  | 100 |  | 60 |  | 50 |
| **Threatened species number (national)** | 50 |  | 66.7 |  | 80 |  | - |
| **Threatened species proportion (global)** | 25 |  | 0 |  | 0 |  | 0 |
| **Threatened species proportion (national)** | 0 |  | 0 |  | 0 |  | - |

**Supplementary Table S5.** Area under each category of sampling effort priority per tetrapod group. Results are shown in km^2^ and percentage of the total surface area of Uruguay, according to curvilinearity of smoothed species accumulation curves (SACs) for each unit. The scale goes from: Null (i.e.: grid-cells where mean slope of the last 10% of SACs was lower or equal to 0.05), ‘Low’ (between 0.05 and 0.1), ‘Medium’ (between 0.1 and 0.5), High (between 0.5 and 1), to Very High (i.e.: grid-cells where no records were found, or where the sampling effort was so low that it was not possible to calculate species accumulation curves).

|  | **Amphibians** | | **Reptiles** | | **Birds** | | **Mammals** | |
| --- | --- | --- | --- | --- | --- | --- | --- | --- |
| **Sampling Priority** | km^2^ | % | km^2^ | % | km^2^ | % | km^2^ | % |
| Null | 589.1 | 0.3 | 303.5 | 0.2 | 7,955.7 | 4.5 | - | - |
| Low | 1,754.1 | 1 | - | - | 8,581.6 | 4.9 | 1,551 | 0.9 |
| Medium | 24,500.6 | 13.9 | 15,855.7 | 9 | 31,389.2 | 17.8 | 26,972.1 | 15.3 |
| High | 14,470.4 | 8.2 | 20,071.3 | 11.4 | 51,929.4 | 29.5 | 23,279.8 | 13.2 |
| Very High | 134,874.4 | 76.6 | 139,958.2 | 79.4 | 76,332.7 | 43.3 | 124,386.0 | 70.6 |
